# Effects of microbial fertilizer and green manure cropping systems on fruit quality of Korla fragrant pear

**DOI:** 10.3389/fpls.2025.1680899

**Published:** 2025-11-06

**Authors:** Yixin Ke, Jie Li, Zhanyi He, Xiuxiu Liu, Xing Shen, Zhongping Chai, Maomao Zeng

**Affiliations:** 1 College of Resources and Environment, Xinjiang Agricultural University, Urumqi, China; 2 Xinjiang Key Laboratory of Soil and Plant Ecological Processes, Xinjiang Agricultural University, Urumqi, China; 3 State Key Laboratory of Food Science and Resources, Jiangnan University, Wuxi, China; 4 School of Food Science and Technology, Jiangnan University, Wuxi, China

**Keywords:** Korla fragrant pear, green manure, flavor, volatile compounds, zinc (Zn), enzymes

## Abstract

Korla Fragrant Pear(Pyrus sinkiangensis Yu), a high-value Geographical Indication product from Xinjiang, China, faces declining fruit quality due to soil degradation from intensive monoculture. This study investigated microbial fertilizer (JF) and green manure (oil sunflower - DK1, DK2; sweet clover - CM1, CM2) intercropping in a pear orchard to improve soil and fruit quality, compared to conventional fertilization (CK). Comprehensive analyses assessed soil properties, fruit minerals, metabolites (monosaccharides, fatty acids, amino acids), and volatile organic compounds (VOCs).Both JF and green manure treatments improved soil physicochemical properties, with CM1 showing the greatest effect. Treatments JF, CM1, CM2, DK1, and DK2 significantly increased fruit K, P, Fe, and Mn content but significantly decreased Zn content. JF significantly enhanced monosaccharide accumulation, while CM1 and high-density oil sunflower (DK2) notably increased fatty acid and free amino acid content. However, all treatments (JF, CM1, CM2, DK1, DK2) significantly reduced total fruit VOCs. Correlation analysis indicated fruit Zn content was positively correlated with most VOCs. Reduced Zn inhibited alcohol dehydrogenase (ADH) and copper-zinc superoxide dismutase (Cu/Zn SOD) activity, leading to decreased VOC synthesis. Therefore, while CM1 is recommended as the optimal strategy for improving basic nutritional quality and soil fertility, coordinated zinc nutrition management is essential when implementing green manure to balance nutritional enhancement with maintaining characteristic flavor, ensuring sustainable industry development.

## 1 Introduction

Korla Fragrant Pear (Pyrus sinkiangensis Yu), a distinctive fruit tree germplasm resource of Xinjiang, has a cultivation history traceable back to the 6th century AD (Chen et al., 2006). It has now become a pillar industry of the agricultural economy in Southern Xinjiang, with a cultivation area exceeding one million mu (Jia et al., 2018; Yu et al., 2022). The fruit quality of this cultivar exhibits multi-dimensional advantages: the synergistic effect of a high sugar-acid ratio (17.47-17.68) and soluble solids content (10.56-10.59%) (Yang et al., 2024) contributes to its sweet and full-bodied flavor; the characteristic of discretely distributed and extremely low content of stone cell clusters (0.315-0.652%) (Wang et al., 2022) imparts a fine-textured flesh; concurrently, the flesh is rich in vitamin C, which supports immune function and promotes collagen synthesis (Qiao et al., 2020; Wang et al., 2022). Furthermore, the dynamic balance of organic acids during ripening, where malic acid accounts for over 80% of the metabolic profile (Yu et al., 2018), further optimizes the fruit softening process. Crucially, its volatile organic compound (VOCs) metabolic network comprises 68 characteristic components (Liu et al., 2023). Among these, esters (ethyl acetate, butyl hexanoate) and aldehydes (hexanal, (E)-2-hexenal) with odor activity values (OAV > 1) form a unique distinct flavor profile, serving as the core driver of quality premium.

To increase yield and income, fruit growers commonly apply inorganic fertilizers. While inorganic fertilizers can rapidly replenish nutrients for crops, excessive application damages soil aggregate structure (Dinesh et al., 2010), reduces soil aeration and permeability (Shi et al., 2024), and consequently severely impedes root respiration and the normal uptake of nutrients (Kong et al., 2017). As these issues have become increasingly prominent, both the yield and quality of Korla fragrant pears have shown varying degrees of decline. To safeguard the quality and yield of Korla fragrant pears, planting green manure and applying microbial fertilizers have emerged as practical and cost-effective soil improvement methods (Otto et al., 2020; Sahgal et al., 2024). As a widely adopted field management practice, green manure cultivation has been proven to offer multiple soil improvement benefits (Hu et al., 2023). The nitrogen-fixing ability of leguminous green manures converts atmospheric nitrogen into plant-available forms, significantly increasing soil nitrogen content (Chen et al., 2014). Simultaneously, green manure can improve soil pH, creating conditions more favorable for crop growth (Kama et al., 2025). Additionally, green manure effectively reduces soil bulk density, enhances soil aeration, and provides a favorable environment for root growth (Glab, 2014). Its organic matter increases soil porosity, optimizes soil structure, and enhances soil aggregate stability (Fang et al., 2021). Furthermore, green manure can improve fruit yield and quality; for instance, Ramirez-Perez et al. found in vineyards that leguminous green manure significantly increased grape yield and soluble solids content (Ramírez-Pérez et al., 2024).Microbial fertilizers, through their rich beneficial microorganisms, play multiple key roles in the soil (Kumar et al., 2025). These microorganisms can activate soil nutrients, converting elements such as nitrogen, phosphorus, and potassium—which are difficult for crops to absorb and utilize—into available forms, thereby providing a continuous supply of nutrition for plants (Pandey and Saharan, 2025). Concurrently, they can secrete substances like organic acids and polysaccharides to improve soil aggregate structure, enhance soil aeration and water retention capacity, and thus promote root development (Guo et al., 2018). Moreover, microorganisms in microbial fertilizers can produce antimicrobial substances and suppress the growth of soil-borne pathogens through competition, enhancing crop disease resistance and stress tolerance (Cucu et al., 2025). Through these mechanisms, microbial fertilizers not only significantly reduce the application of chemical fertilizers, mitigating soil degradation and environmental pollution, but also help improve fruit yield and quality, making them an important measure for achieving sustainable agricultural development. Therefore, planting green manure and applying microbial fertilizers are effective field management measures for improving the soil environment in Korla fragrant pear orchards and enhancing pear quality and yield.

Previous studies have predominantly focused on the analysis of characteristic aroma components, paying insufficient attention to soil degradation issues in pear orchards. Addressing the problem of flavor deterioration in Korla Fragrant Pear fruit caused by soil degradation in orchards, this study conducted a field intercropping experiment with green manure in a 7-8-year-old pear orchard to investigate its impact on Korla Fragrant Pear quality. The research aims to provide theoretical foundations and technical pathways for green manure-driven soil improvement and flavor enhancement in pear orchards. This is of significant practical importance for facilitating a paradigm shift in the Korla fragrant pear industry from “yield priority” towards “quality orientation”.

## 2 Materials and methods

### 2.1 Experimental site description

The experiment was conducted during 2023 in Heshilike Township, Korla City, Bayingolin Mongol Autonomous Prefecture, Xinjiang, located at coordinates 41°43’38”N, 85°57’46”E with an elevation of 855.3 meters. Situated in central Xinjiang, the site lies at the southern foothills of the Tianshan Mountains on the northeastern edge of the Tarim Basin. It is bordered by the Tianshan branch range to the north and adjacent to the Taklimakan Desert, the world’s second-largest desert, to the south. The region experiences a warm-temperate continental climate characterized by significant diurnal temperature variations and abundant sunshine. The average annual sunshine duration is 2990 hours. The mean annual temperature ranges between 14 and 15°C, while annual precipitation falls between 50 and 58 millimeters. The maximum annual evaporation reaches 2788.2 millimeters. The effective accumulated temperature (≥10°C) ranges from 4100 to 4400°C, and the frost-free period lasts 210 to 239 days. The prevailing wind direction is from the northeast. The orchard soil has a sandy texture with the following baseline nutrient levels, pH 7.80, soil organic matter (SOM) 11.22 grams per kilogram, available phosphorus (AP) 12.06 milligrams per kilogram, available potassium (AK) 167.17 milligrams per kilogram, and alkali-hydrolyzable nitrogen (AN) 16.63 milligrams per kilogram.

### 2.2 Plant material and experimental design

The study was carried out over the 2022 to 2023 period using 7 - 8-year-old Korla Fragrant Pear (Pyrus sinkiangensis Yu) trees grafted onto Pyrus betulifolia rootstock. Trees were spaced at 3 meters by 5 meters, equivalent to 675 trees per hectare. Six fertilization treatments were implemented within the pear orchard. These treatments included conventional fertilization (CK), application of microbial fertilizer (JF) containing Bacillus subtilis and Bacillus licheniformis, intercropping with oil sunflower (Helianthus annuus L) at a low density (DK1, row spacing 25 centimeters, seeding rate 27 kilograms per hectare), intercropping with oil sunflower at a high density (DK2, row spacing 20 centimeters, seeding rate 33 kilograms per hectare), intercropping with sweet clover (Melilotus officinalis Linn.) at a low density (CM1, row spacing 25 centimeters, seeding rate 21 kilograms per hectare), and intercropping with sweet clover at a high density (CM2, row spacing 20 centimeters, seeding rate 27 kilograms per hectare). This constituted a field trial with different fertilization treatments applied during the growing season. Details regarding fertilizer application rates, specific green manure species, and sowing techniques are presented in **Table 1**.

**Table 1:** Experimental treatment design plan.

Treatment	Green manure type	Seeding rate (kg/hm^2^)	Sowing depth (cm)	Row spacing (cm)	Agricultural microbial fertilizer(kg/hm^2^)	Nutrient application ratekg/hm (Jia et al., 2018)		
						N	P_2_O_5_	K_2_O
CK	None	0	0	0	0	300	300	150
JF	None	0	0	0	1200	300	300	150
DK1	Oil Sunflower	27	2-3	25	0	300	300	150
DK2	Oil Sunflower	33	2-3	20	0	300	300	150
CM1	Sweetclover	21	1	25	0	300	300	150
CM2	Sweetclover	27	1	20	0	300	300	150

Each treatment plot covered an area of 666.67 kg per hectare, with three replicates per treatment, resulting in a total experimental area of 12,000 kg per hectare. A base application of sheep manure at 15,000 kilograms per hectare was applied once during autumn. Phosphorus fertilizer applied as triple superphosphate (46% P_2_O_5_) and potassium fertilizer applied as potassium sulfate (51% K_2_O) were fully applied before the spring bud break stage. Nitrogen fertilizer applied as urea (46% N) and the microbial fertilizer (Shipulang produced by Tsuneishi Fertilizer (Qingdao) Co., Ltd., containing ≥500 million CFU per gram of Bacillus subtilis and Bacillus licheniformis) were applied in split doses. Sixty percent of the nitrogen and microbial fertilizer was applied before bud break, with the remaining forty percent applied as a top dressing at the early fruit expansion stage. Fertilization involved digging an annular trench approximately 30 centimeters deep and 30 centimeters wide, located 50 to 80 centimeters from the central trunk. Fertilizers were evenly distributed within this trench. Sweet clover and oil sunflower were used as green manure crops. Seeds sourced from the Inner Mongolia Autonomous Region were purchased from Gansu Lanbin Ecological Technology Co., Ltd. Sweet clover seeds had 95% purity and an 85% germination rate. Oil sunflower seeds had 90% purity and an 80% germination rate. The distance between the pear tree rows and the green manure rows was maintained at 70 to 80 centimeters. Green manure sowing occurred in early April, followed by mechanical crushing and soil incorporation in late July.

### 2.3 Sample collection and processing

Soil samples were collected during the Korla Fragrant Pear ripening period on September 6, 2023, within the orchard. For each treatment, five healthy pear trees exhibiting similar growth vigor were selected and tagged using the five-point sampling method. Soil samples from the 0–20 centimeter layer were collected from both sides of the fertilization trench after removing surface litter. Soil samples from the same depth on both sides of the trench for each replicate tree were combined into one composite sample. After initial mixing and breaking of clods, soil samples were placed in zip-lock bags, stored in insulated containers with dry ice, and transported to the laboratory under refrigerated conditions. Upon returning to the laboratory, soil samples were air-dried. Plant roots and stones were removed by sieving the soil through a 2-millimeter mesh. The sieved soil was thoroughly homogenized by further sieving through a 1-millimeter mesh and stored in sealed bags for physicochemical analysis.

Fruit sampling coincided with soil collection. From each of the five tagged trees per treatment, 12 mature fruits were harvested from the mid-upper canopy (1.5 to 2.0 meters above ground level) in the east, south, west, and north directions, resulting in 60 fruits collected per treatment. Fruits were rinsed with ultrapure water. After coring with a ceramic knife, the flesh was sliced into 5-millimeter pieces. The flesh slices were homogenized using a blender. The homogenate was filtered through a 100-mesh nylon sieve. The filtered homogenate was aliquoted into 5-milliliter acid-washed centrifuge tubes and stored at -80°C ultra-low temperature freezers for subsequent analysis of mineral elements, monosaccharides, free amino acids, fatty acids, and volatile compounds.

### 2.4 Analytical methods

Soil physicochemical properties were analyzed following methods described in Bao’s Soil and Agricultural Chemistry Analysis (Bao, 2000). Soil pH was measured potentiometrically using a pH meter with a soil-to-water ratio of 2.5 to 1. Electrical conductivity (EC) was determined using a conductivity meter. Total nitrogen (TN) content was measured using the semi-micro Kjeldahl method. Soil organic matter (SOM) content was determined by the potassium dichromate external heating method. Alkali-hydrolyzable nitrogen (AN) was analyzed using the alkaline hydrolysis diffusion method. Available phosphorus (AP) was extracted using sodium bicarbonate and quantified by the molybdenum antimony anti-spectrophotometric method. Available potassium (AK) was extracted using ammonium acetate and measured by flame photometry.

Monosaccharide composition was analyzed using a Thermo ICS5000+ ion chromatography system (Thermo Fisher Scientific, Massachusetts, USA) according to the method described by He et al. (2018) (He et al., 2018).

Amino acid analysis was performed using a Waters 2695 high-performance liquid chromatography (HPLC) system (Waters Corporation, Massachusetts, USA) following the method of Chen et al. (2015) (Chen et al., 2015).

Fatty acid analysis was conducted using hydrolysis-extraction and esterification methods as specified in the Chinese National Standard GB 5009.168—2016.

Analysis of mineral elements in fruit was performed according to the Chinese National Standard GB 5009.268-2016.

Volatile organic compounds were analyzed by headspace solid-phase microextraction coupled with gas chromatography-mass spectrometry (HS-SPME-GC-MS). Five grams of pear homogenate were weighed into a 20-milliliter headspace vial. Five milliliters of saturated sodium chloride (NaCl) solution were added, followed by 10 microliters of cyclohexanone solution (0.947 milligrams per milliliter in chromatographic grade methanol) as an internal standard. The vial was immediately sealed with a polytetrafluoroethylene (PTFE) septum. A pre-conditioned SPME fiber was exposed to the vial headspace at 50°C for 31 minutes to adsorb volatiles. The fiber was then inserted into the GC injector port and thermally desorbed for 7 minutes.

Gas chromatography was performed using an SH-Wax capillary column (30 meters length, 0.25 millimeters internal diameter, 0.25 micrometers film thickness). The injector temperature was set to 250°C in splitless mode. The column temperature program was initiated at 40°C and held for 3 minutes. The temperature was then ramped to 100°C at a rate of 6°C per minute, followed by a ramp to 230°C at 10°C per minute, with a final hold time of 5 minutes. Helium was used as the carrier gas at a constant flow rate of 0.8 milliliters per minute.

Mass spectrometry detection utilized electron ionization (EI) mode at 70 electron volts. Data acquisition was performed in scan mode over a mass range of 33 to 400 mass-to-charge ratio (m/z). The ion source temperature was maintained at 200°C, and the transfer line temperature was set to 250°C. A solvent delay of 1 minute was applied.


$$VOC\;content(mg/L)=\frac{peak\;area\times \;internal\;standard\;concentration}{sample\;weight\times internal\;standard\;peak\;area}$$

### 2.5 Data analysis

All experiments were performed with three biological replicates. Data are presented as mean ± standard deviation (SD). One-way analysis of variance (ANOVA) was conducted using IBM SPSS Statistics 27 software (IBM Corp., Armonk, NY, USA) to determine significant differences (p< 0.05). Cluster analysis and Spearman’s rank correlation coefficient analysis were performed using OriginPro 2024 software (OriginLab Corporation, Northampton, MA, USA).

## 3 Results and analysis

### 3.1 Effects of different organic fertilizers on soil physicochemical properties in Korla fragrant pear orchards

Data presented in **Table 2** show that soil pH and electrical conductivity (EC) in all treatment groups were significantly lower than those in the CK treatment (*p<* 0.05). Compared to CK, soil pH decreased by 2.55%, 4.02%, 2.95%, 4.02%, and 2.68% in the JF, CM1, CM2, DK1, and DK2 treatments, respectively. Among these, the CM1 and DK1 treatments showed the largest pH reduction (4.02% lower than CK), which was significantly greater than the reductions observed in the other treatments. This indicates that low-density planting of sweet clover and oil sunflower green manures significantly reduced soil pH. Soil EC decreased by 16.02%, 20.48%, 9.15%, and 9.27% in the JF, CM1, CM2, and DK1 treatments compared to CK, with the JF and CM1 treatments demonstrating the most significant reductions in EC. In contrast, soil organic matter (SOM) content was significantly increased in all treatments compared to CK (*p<* 0.05). The increases were 12.02% for JF, 15.42% for CM1, 12.85% for CM2, 24.32% for DK1, and 17.33% for DK2. The green manure treatments (DK and CM series) generally exhibited higher SOM content than the microbial fertilizer treatment (JF), with the DK1 treatment showing the most pronounced effect.

**Table 2:** Soil physicochemical properties under different green manure incorporation treatments in Korla fragrant pear orchards.

Treatment	pH	EC	SOM	AK	AP	AN
CK	7.46 ± 0.08a	1373.33 ± 4.04a	10.87 ± 0.52c	161.33 ± 7.51c	11.11 ± 0.25c	16.25 ± 0.44d
JF	7.27 ± 0.01b	1153.33 ± 48.17c	12.17 ± 0.39b	178.00 ± 2.65b	12.9 ± 0.35b	18.80 ± 0.18b
CM1	7.16 ± 0.03c	1092.00 ± 67.74c	12.55 ± 0.06b	188.00 ± 2.00a	14.04 ± 0.30a	20.00 ± 0.39a
CM2	7.26 ± 0.02b	1247.67 ± 50.64b	12.26 ± 0.19b	176.67 ± 8.02b	13.79 ± 0.41a	19.05 ± 0.36b
DK1	7.16 ± 0.03c	1246.00 ± 21.93b	13.51 ± 0.24a	189.67 ± 3.22a	13.88 ± 0.26a	18.80 ± 0.18b
DK2	7.24 ± 0.07bc	1296.67 ± 56.01ab	12.76 ± 0.54b	171.33 ± 5.77b	13.74 ± 0.15a	17.88 ± 0.35c

SOM, Soil organic matter (g/kg); AN, Alkali-hydrolyzable nitrogen (mg/kg); AP, Available phosphorus (mg/kg); AK, Available potassium (mg/kg).

Data are presented as mean ± standard deviation (n = 3), with a significance level of p = 0.05. Different lowercase letters in the same column indicate significant differences (*p<* 0.05).

Regarding available nutrients, the contents of available nitrogen (AN), available phosphorus (AP), and available potassium (AK) were all significantly higher in all treatment groups compared to CK (*p<* 0.05). Soil AN increased significantly by 15.69%, 15.69%, 10.03%, 23.08%, and 17.23% in the JF, CM1, CM2, DK1, and DK2 treatments, respectively, compared to CK. The DK series, particularly DK1, showed the greatest increase, suggesting that planting green manure, especially oil sunflower at low density, offers a greater advantage in enhancing soil AN. Soil AP increased significantly by 16.11%, 24.93%, 23.67%, 26.37%, and 24.12% in the JF, CM1, CM2, DK1, and DK2 treatments, respectively. The improvement in AP was more substantial with green manure cultivation than with microbial fertilizer application, and no significant difference was observed between planting oil sunflower and sweet clover. Soil AK content increased by 10.33%, 17.57%, 6.20%, 16.53%, and 9.51% in the JF, CM1, CM2, DK1, and DK2 treatments, respectively. The largest increases in AK were observed in the DK1 (16.53%) and CM1 (17.57%) treatments, which were significantly superior to the other treatments. In summary, low-density sweet clover (CM1) demonstrated the most prominent effect in improving soil physicochemical properties, significantly outperforming the other treatments.

### 3.2 Effects of different organic fertilizers on mineral element content in Korla fragrant pears

**Figure 1:**
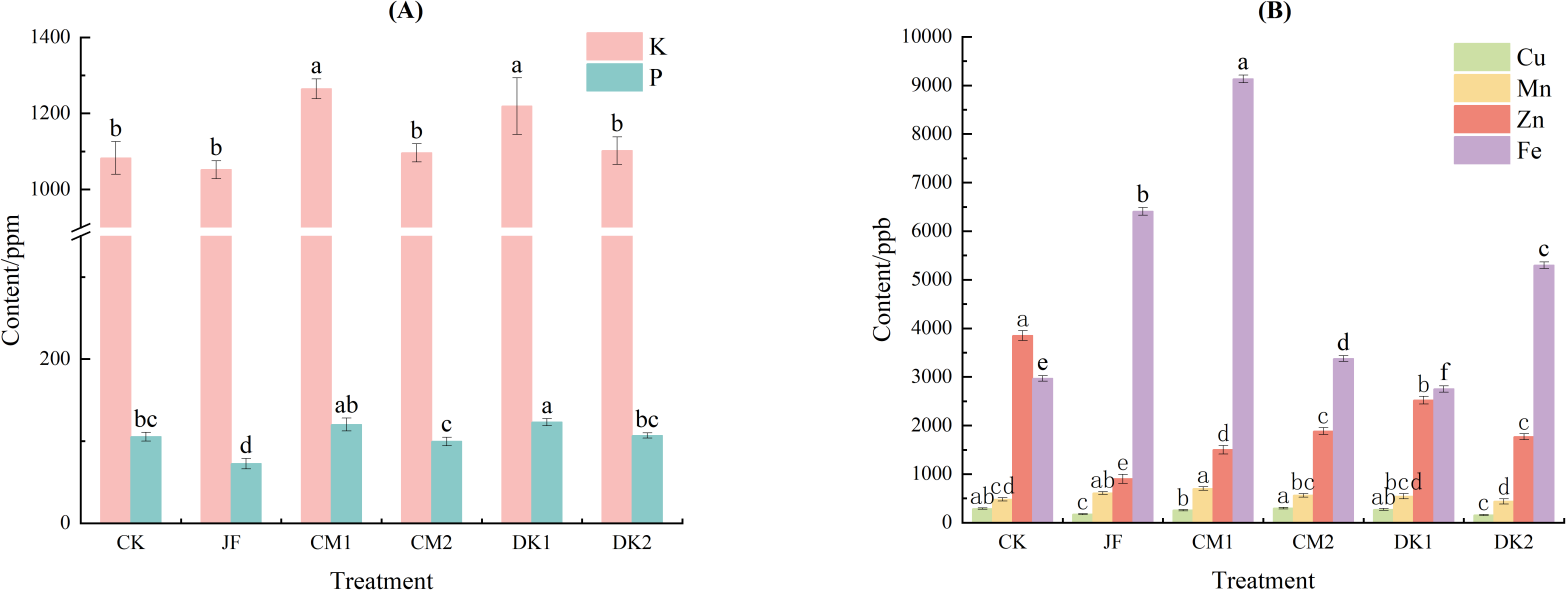
The effects of incorporating different green manures on K,P **(A)**, and trace elements **(B)** in Korla fragrant pears. Data are presented as mean ± standard deviation (n = 3), with a significance level of P = 0.05. Different lowercase letters in the same column indicate significant differences (*P<* 0.05).

Different organic fertilizers exerted varied regulatory effects on the mineral element content of Korla Fragrant Pears (**Figure 1A**). Compared to the CK treatment, the JF treatment reduced fruit potassium (K) content by 2.7%. In contrast, all green manure treatments increased fruit K content. The CM1 and DK1 treatments showed significant increases of 16.8% and 12.6%, respectively, compared to CK (*p<* 0.05). This trend aligned with the changes observed in soil available potassium content. The JF treatment significantly decreased fruit phosphorus (P) content (*p<* 0.05). Conversely, the CM1 and DK1 treatments enhanced fruit P content, increasing it by 14.10% and 16.86%, respectively. Among these, only the DK1 treatment showed a significant difference compared to CK (*p<* 0.05).

Regarding trace elements (**Figure 1B**), fruit iron (Fe) content increased significantly compared to CK in the JF, CM1, CM2, and DK2 treatments (*p<* 0.05), with increases of 115.9%, 207.7%, 13.8%, and 78.5%, respectively. The CM1 treatment resulted in significantly higher Fe content than the JF, CM2, and DK2 treatments. Conversely, the DK1 treatment led to a significant 7.3% reduction in Fe content (*p<* 0.05). Fruit manganese (Mn) content increased significantly in the JF and CM1 treatments (*p<* 0.05), rising by 26.6% and 46.2%, respectively, compared to CK. No significant differences in Mn content were observed between the CM2, DK1, DK2 treatments and CK. Fruit copper (Cu) content decreased significantly in the JF and DK2 treatments by 39.8% and 46.1%, respectively, compared to CK (*p<* 0.05). The CM1, CM2, and DK1 treatments showed no significant difference in Cu content compared to CK. Overall, Cu levels in fruit from green manure treatments remained stable, indicating that green manure cultivation helped maintain copper homeostasis in the fruit. All treatments (JF, CM1, CM2, DK1, DK2) significantly reduced fruit zinc (Zn) content compared to CK (*p<* 0.05), with reductions of 61.9%, 34.5%, 54.0%, 55.8%, and 37.6%, respectively. The Zn content in fruit from the JF treatment was significantly lower than that from the green manure treatments. Within the green manure treatments, Zn content followed the order CM1 > CM2 and DK1 > DK2.

### 3.3 Effects of different organic fertilizers on monosaccharides in Korla fragrant pears

**Figure 2:**
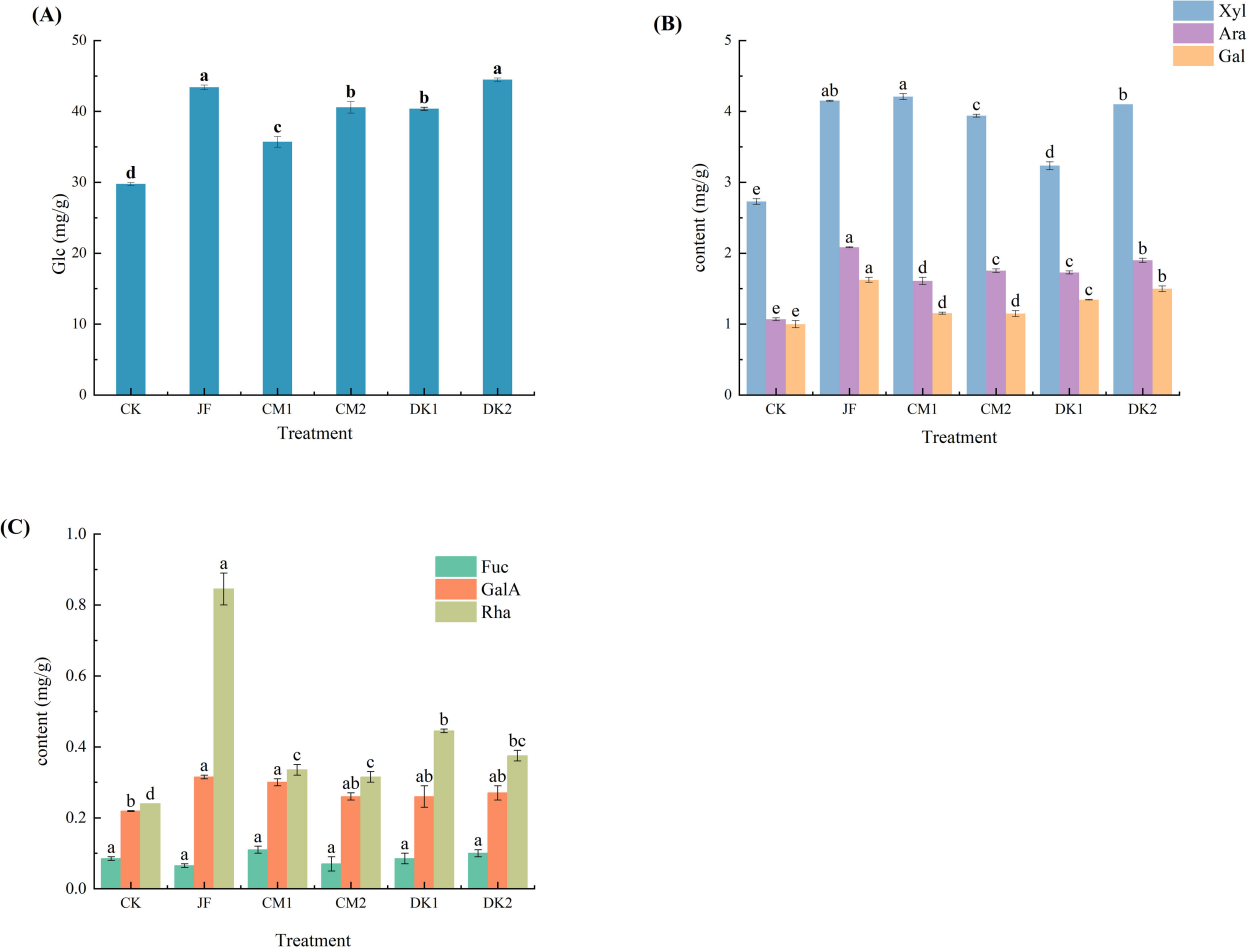
The effects of incorporating different green manures on monosaccharides in Korla fragrant pears. Data are presented as mean ± standard deviation (n = 3), with a significance level of P = 0.05. Different lowercase letters in the same column indicate significant differences (*P<* 0.05).

The results demonstrate that organic fertilizer treatments significantly altered the monosaccharide composition in Korla Fragrant Pear fruit (**Figure 2**). Among the seven monosaccharides analyzed, compared to the CK treatment, all organic fertilizer treatments (JF, CM1, CM2, DK1, DK2) upregulated the accumulation levels of glucose, arabinose, galactose, xylose, galacturonic acid, and rhamnose. Fucose levels remained stable across all treatments.

Regarding the dominant sugar glucose (**Figure 2A**), significant increases were observed in the JF, CM1, DK1, and DK2 treatments compared to CK (*p<* 0.05), with increases of 29.99%, 27.04%, 21.31%, and 39.26%, respectively. The DK2 treatment showed the most pronounced effect. Pentose sugar accumulation exhibited differential responses (**Figure 2B**). Arabinose content, a pentose sugar, was significantly elevated by all treatments (JF, CM1, CM2, DK1, DK2) compared to CK (*p<* 0.05), with increases of 94.86%, 50.47%, 64.02%, 61.68%, and 77.57%, respectively. The JF and DK2 treatments yielded the highest increases. Galactose content was also significantly increased by all treatments (*p<* 0.05), rising by 62.50%, 15.50%, 15.00%, 34.50%, and 50.00% in the JF, CM1, CM2, DK1, and DK2 treatments, respectively, compared to CK. Xylose content, another pentose sugar, was significantly enhanced by all treatments (*p<* 0.05), increasing by 52.05%, 54.21%, 44.32%, 18.50%, and 50.18% in the JF, CM1, CM2, DK1, and DK2 treatments, respectively. The JF and CM1 treatments were significantly more effective than the others in boosting xylose.

Galacturonic acid content was significantly increased by the JF and CM1 treatments (*p<* 0.05), rising by 43.84% and 36.99% compared to CK, with the JF treatment showing the greater effect(**Figure 2C**). Rhamnose content was significantly elevated by all treatments (*p<* 0.05), increasing by 192.62%, 132.79%, 29.10%, 61.07%, and 54.51% in the JF, CM1, CM2, DK1, and DK2 treatments, respectively. The JF and CM1 treatments were significantly superior to the others in enhancing rhamnose. Notably, fucose content showed no significant differences among the treatments, ranging from 0.065 to 0.110. In summary, the efficacy of different organic fertilizer treatments in upregulating specific monosaccharides varied. The DK2 treatment was optimal for glucose enhancement. The JF treatment significantly enhanced arabinose, galactose, rhamnose, and galacturonic acid. The CM1 treatment was relatively more effective for xylose accumulation. Fucose metabolism remained insensitive to the organic fertilizer treatments.

### 3.4 Effects of different organic fertilizers on free amino acids in Korla fragrant pears

The results demonstrate that different treatments significantly modulated amino acid metabolism (**Table 3**). Among the umami amino acids, the JF, CM1, CM2, and DK2 treatments significantly increased the content of aspartic acid (Asp) in the fruit compared to CK (*p<* 0.05), with increases of 35.26%, 82.92%, 29.48%, and 52.76%, respectively. The CM1 treatment exhibited the strongest accumulation, significantly outperforming the other treatments. The JF, CM1, and DK2 treatments significantly increased glutamic acid (Glu) content, rising by 16.62%, 16.88%, and 11.86% compared to CK (*p<* 0.05). For umami amino acids, the CM1 treatment yielded the most favorable results.

**Table 3:** The effects of incorporating different green manures on the content of free amino acids in Korla fragrant pears (*μ*g/kg).

Free Amino acid	CK	JF	CM1	CM2	DK1	DK2
asp	33.55 ± 0.99d	45.38 ± 0.43c	61.37 ± 0.65a	43.44 ± 0.17c	31.7 ± 1.02d	51.25 ± 1.99b
glu	7.76 ± 0.04b	9.05 ± 0.06a	9.07 ± 0.06a	7.49 ± 0.04b	7.37 ± 0.26b	8.68 ± 0.23a
ser	1.47 ± 0.10d	2.46 ± 0.10b	2.00 ± 0.08c	2.33 ± 0.12b	1.94 ± 0.04c	2.84 ± 0.05a
his	0.38 ± 0.08b	0.48 ± 0.02b	0.51 ± 0.06b	0.57 ± 0.13b	0.42 ± 0.04b	1.38 ± 0.07a
gly	0.49 ± 0.20a	0.6 ± 0.03a	0.41 ± 0.03a	0.56 ± 0.19a	0.36 ± 0.02a	0.56 ± 0.03a
thr	3.51 ± 0.03d	6.59 ± 0.06b	4.6 ± 0.04c	4.42 ± 0.02c	4.42 ± 0.12c	7.29 ± 0.15a
arg	0.73 ± 0.20a	0.93 ± 0.34a	0.56 ± 0.08a	0.54 ± 0.05a	0.51 ± 0.11a	0.5 ± 0.11a
ala	3.89 ± 0.11e	5.76 ± 0.13b	5.15 ± 0.10c	4.55 ± 0.01d	4.34 ± 0.23d	6.62 ± 0.03a
tyr	0.54 ± 0.08b	0.89 ± 0.23a	0.41 ± 0.08b	0.4 ± 0.03b	0.45 ± 0.18b	0.49 ± 0.14b
cys-s	0.45 ± 0.00b	0.8 ± 0.05a	0.86 ± 0.03a	0.86 ± 0.03a	0.52 ± 0.00b	0.93 ± 0.00a
val	8.15 ± 0.12d	12.77 ± 0.00b	8.91 ± 0.07c	8.94 ± 0.06c	8.84 ± 0.28c	15.07 ± 0.24a
met	0.69 ± 0.02c	1.16 ± 0.00b	0.97 ± 0.02b	1.13 ± 0.09b	1.04 ± 0.02b	2.12 ± 0.07a
phe	0.44 ± 0.02a	0.53 ± 0.12a	0.47 ± 0.01a	0.51 ± 0.09a	0.47 ± 0.03a	0.46 ± 0.03a
ile	2.76 ± 0.01e	4.55 ± 0.07b	3.06 ± 0.04cd	2.92 ± 0.07de	3.28 ± 0.07c	5.72 ± 0.14a
leu	0.54 ± 0.04c	0.82 ± 0.08b	0.61 ± 0.00bc	0.65 ± 0.06bc	0.53 ± 0.02c	1.34 ± 0.05a
lys	0.28 ± 0.08a	0.46 ± 0.24a	0.23 ± 0.00a	0.29 ± 0.04a	0.21 ± 0.01a	0.28 ± 0.03a
pro	2.10 ± 0.32b	2.53 ± 0.39ab	2.40 ± 0.11ab	1.99 ± 0.10b	2.23 ± 0.31ab	2.93 ± 0.29a

Data are presented as mean ± standard deviation (n = 3), with a significance level of p = 0.05. Different lowercase letters in the same column indicate significant differences (*p<* 0.05).

Regarding sweet amino acids, the JF, CM1, CM2, and DK2 treatments significantly increased serine (Ser) content compared to CK (*p<* 0.05), with increases of 67.35%, 36.05%, 58.50%, and 93.20%, respectively. The DK2 treatment was significantly superior to the others. All treatments (JF, CM1, CM2, DK1, DK2) significantly increased alanine (Ala) content (*p<* 0.05), showing increases of 48.07%, 32.39%, 16.97%, 11.57%, and 70.18% compared to CK. The DK2 treatment demonstrated significantly greater efficacy than the other treatments. Sweet amino acid accumulation was strongest under the DK2 treatment.

For essential amino acids, all treatments (JF, CM1, CM2, DK1, DK2) significantly increased threonine (Thr) content compared to CK (*p<* 0.05), with increases of 87.75%, 31.05%, 25.93%, 25.93%, and 107.69%, respectively. The DK1 treatment showed a relative advantage over others for Thr. Valine (Val) content was significantly increased by all treatments (*p<* 0.05), rising by 56.69%, 9.33%, 9.69%, 8.47%, and 84.91% in the JF, CM1, CM2, DK1, and DK2 treatments, respectively, with DK2 yielding the highest Val content. Methionine (Met) content was significantly elevated by all treatments (*p<* 0.05), increasing by 68.12%, 40.58%, 63.77%, 50.72%, and 207.25% in the JF, CM1, CM2, DK1, and DK2 treatments, respectively. Isoleucine (Ile) content was significantly increased by all treatments (*p<* 0.05), showing gains of 64.86%, 10.87%, 5.80%, 18.84%, and 107.25% compared to CK. The DK2 treatment resulted in significantly higher Ile content than the other groups. Leucine (Leu) content was significantly increased by the JF, CM1, CM2, and DK2 treatments (*p<* 0.05), with increases of 51.85%, 12.96%, 20.37%, and 148.15% compared to CK. The DK2 treatment induced the most significant accumulation of Leu. Overall, the DK2 treatment demonstrated the most substantial increases in the majority of essential amino acids (Thr, Val, Met, Ile, Leu).

Among other amino acids, all treatments (JF, CM1, CM2, DK1, DK2) significantly increased histidine (His) content compared to CK (*p<* 0.05), with increases of 26.32%, 34.21%, 50.00%, 10.53%, and 263.16%, respectively. The accumulation under the DK2 treatment was exceptionally pronounced. This study demonstrates that organic fertilizer application enhances free amino acid content in Korla Fragrant Pears. Further analysis indicates that the DK2 treatment exhibited the most significant effect on amino acid accumulation. This treatment appears to promote the synthesis and accumulation of amino acids through specific metabolic pathways, playing a pivotal role in the overall regulation of amino acid metabolism.

### 3.5 Effects of different organic fertilizers on fatty acids in Korla fragrant pears

This study revealed the differential regulatory mechanisms of green manure treatments on fatty acid metabolism in Korla Fragrant Pear fruit (**Table 4**). The influence of different treatments on the content of various fatty acids exhibited significant differences. Among short-chain fatty acids, all treatments (JF, CM1, CM2, DK1, DK2) significantly increased the content of C10:0 (decanoic acid) compared to CK (*p<* 0.05), with increases of 52.54%, 240.34%, 133.22%, 148.47%, and 76.61%, respectively. The CM1 treatment showed the strongest accumulation, significantly outperforming the other treatments (*p<* 0.05).

**Table 4:** The effects of incorporating different green manures on the fatty acid content in Korla fragrant pears (*μ*g/g).

Fatty acid	CK	JF	CM1	CM2	DK1	DK2
C10:0	0.003 ± 0.001e	4.500 ± 0.640de	10.040 ± 0.010a	6.880 ± 0.720bc	7.330 ± 0.710b	5.210 ± 0.680cd
C12:0	0.023 ± 0.010a	24.720 ± 2.310a	21.990 ± 5.790a	20.820 ± 4.170a	26.600 ± 3.790a	18.430 ± 2.010a
C16:0	0.149 ± 0.035b	153.350 ± 1.700ab	235.920 ± 1.180a	190.220 ± 20.850ab	186.380 ± 63.730ab	236.620 ± 5.280a
C17:0	0.07 ± 0.014a	80.660 ± 1.380a	92.700 ± 10.380a	86.170 ± 29.390a	91.430 ± 8.130a	80.750 ± 2.470a
C17:1	0.141 ± 0.061a	284.740 ± 34.610a	274.230 ± 126.010a	247.270 ± 130.900a	150.130 ± 7.670a	160.100 ± 27.710a
C18:0	0.079 ± 0.008a	79.030 ± 0.930a	112.450 ± 0.800a	89.230 ± 6.950a	96.830 ± 32.260a	108.630 ± 6.110a
C18:1,c+t	0.011 ± 0.003c	12.180 ± 0.080bc	19.050 ± 0.170ab	14.040 ± 0.900bc	12.470 ± 5.240bc	23.630 ± 0.240a
C18:2,trans	0.104 ± 0.038c	114.97 ± 1.17bc	181.270 ± 1.780ab	144.720 ± 23.990abc	128.720 ± 44.470abc	193.530 ± 1.020a
C18:3n3	0.008 ± 0.003c	10.69 ± 0.03bc	14.320 ± 0.090b	11.350 ± 1.690bc	10.720 ± 3.710bc	21.090 ± 0.200a
C20:0	0.004 ± 0.00b	4.110 ± 0.220ab	5.450 ± 0.600ab	5.040 ± 0.830ab	4.120 ± 1.880ab	6.150 ± 0.500a
C22:2	0.026 ± 0.01b	28.780 ± 1.620b	49.810 ± 4.190a	35.070 ± 0.370b	38.460 ± 6.710ab	31.630 ± 2.010b
C24:1	0.01 ± 0.003c	11.640 ± 1.780c	25.320 ± 1.680a	15.900 ± 0.650bc	17.650 ± 3.870b	15.630 ± 0.650bc

Data are presented as mean ± standard deviation (n = 3), with a significance level of p = 0.05. Different lowercase letters in the same column indicate significant differences (*p<* 0.05).

For medium-chain fatty acids, the JF and DK1 treatments significantly increased the content of C12:0 (lauric acid) compared to CK (*p<* 0.05), rising by 9.14% and 17.44%, respectively. The DK1 treatment induced the strongest accumulation. All treatments significantly increased the content of C16:0 (palmitic acid) (*p<* 0.05), showing gains of 2.65%, 57.92%, 27.33%, 24.76%, and 58.39% in the JF, CM1, CM2, DK1, and DK2 treatments, respectively, compared to CK. The DK2 treatment yielded the strongest accumulation of palmitic acid (*p<* 0.05).

Regarding long-chain monounsaturated fatty acids, all treatments significantly increased the content of C18:1,c+t (cis + trans oleic acid) (*p<* 0.05), rising by 13.94%, 78.20%, 31.34%, 16.65%, and 121.05% in the JF, CM1, CM2, DK1, and DK2 treatments, respectively, compared to CK. The DK2 treatment was the most effective. All treatments also significantly increased the content of C24:1 (nervonic acid) (*p<* 0.05), with increases of 12.46%, 144.64%, 53.62%, 70.53%, and 51.01%, respectively. The CM1 treatment was the most effective for nervonic acid accumulation.

For long-chain polyunsaturated fatty acids, all treatments significantly increased the content of C18:2,trans (trans-linoleic acid) (*p<* 0.05), showing increases of 10.64%, 74.43%, 39.26%, 23.88%, and 86.25% in the JF, CM1, CM2, DK1, and DK2 treatments, respectively, compared to CK. The DK2 treatment demonstrated the strongest accumulation effect. The content of C18:3n3 (α-linolenic acid) was significantly elevated by all treatments (*p<* 0.05), increasing by 40.29%, 87.93%, 48.95%, 40.68%, and 176.77% in the JF, CM1, CM2, DK1, and DK2 treatments, respectively. The DK2 treatment exhibited the greatest accumulation capacity for α-linolenic acid.

Among long-chain saturated fatty acids, all treatments significantly increased the content of C20:0 (arachidic acid) (*p<* 0.05), with increases of 12.60%, 49.32%, 38.08%, 12.88%, and 68.49% in the JF, CM1, CM2, DK1, and DK2 treatments, respectively, compared to CK. The DK2 treatment showed the strongest accumulation effect. The content of C22:2 (docosadienoic acid) was significantly increased by all treatments (*p<* 0.05), rising by 9.26%, 89.10%, 33.14%, 46.01%, and 20.08% in the JF, CM1, CM2, DK1, and DK2 treatments, respectively. The CM1 treatment induced the strongest accumulation of docosadienoic acid.

Overall, the CM1 and DK2 treatments demonstrated strong accumulation capabilities for the majority of fatty acid components, particularly for C10:0 (decanoic acid), C16:0 (palmitic acid), C18:1,c+t (oleic acid), C18:2,trans (trans-linoleic acid), C18:3n3 (α-linolenic acid), C20:0 (arachidic acid), C22:2 (docosadienoic acid), and C24:1 (nervonic acid). This indicates that these two treatments significantly promote the synthesis and accumulation of fatty acids.

### 3.6 Effects of different organic fertilizers on volatile compounds in Korla fragrant pears

**Figure 3:**
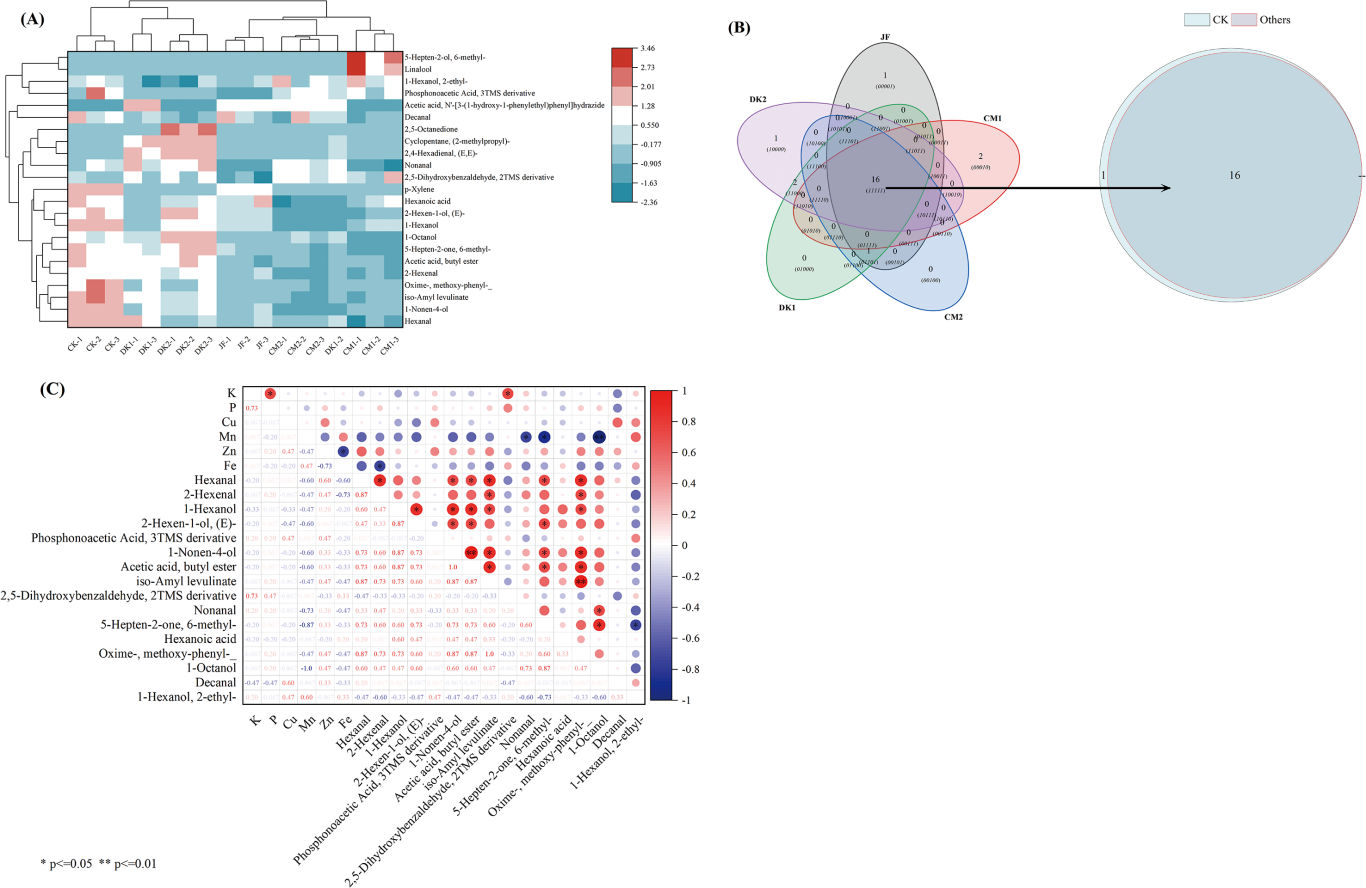
Cluster analysis of VOCs in Korla fragrant pears **(A)**, Venn Diagram **(B)** and Correlation Analysis **(C)**. Data are presented as mean ± standard deviation (n = 3), with a significance level of P = 0.05. Different lowercase letters in the same column indicate significant differences (P< 0.05).

Cluster analysis (**Figure 3A**) revealed good reproducibility among parallel samples within each treatment group (JF, CM1, CM2, DK1, DK2). Compared to the CK treatment, all organic fertilizer treatments (JF, CM1, CM2, DK1, DK2) resulted in a significant reduction in the total volatile compound content in Korla Fragrant Pears. However, under the DK2 treatment specifically, several individual compounds exhibited significantly higher content than other treatments. These included Nonanal, 1-Nonen-4-ol, 1-Octanol, 2,5-Dihydroxybenzaldehyde 2TMS derivative, and 6-Methyl-5-hepten-2-one. Venn analysis (**Figure 3B**) identified 16 core VOCs common to all five treatments. These core VOCs overlapped with those found in CK and showed a significant positive correlation with zinc (Zn) content (**Figure 3C**). Notably, while Zn content decreased by 34.5% - 76.61% across all treatment groups compared to CK, the magnitude of VOC reduction did not exhibit a linear relationship with Zn loss. This observation implied that Zn influences metabolic pathways primarily through the regulation of enzyme activity rather than solely via concentration-dependent mechanisms. The overall volatile content decreased significantly by 7.27% - 58.29% in the treated groups compared to CK. Aldehydes decreased by 1.40% - 60.08%, with specific aldehydes like Hexanal and Nonanal showing significant reductions of 2.45% - 61.46%. Alcohol content also decreased by 14.80%- 77.32%, while the total ester content declined by 14.31% - 59.47%.

## 4 Discussion

### 4.1 Soil physicochemical properties in Korla fragrant pear orchards

Organic fertilizers, particularly microbial fertilizers and green manures, play a crucial role in improving orchard soil physicochemical properties. This is achieved primarily through the microbially mediated humification of their organic matter, which enhances nutrient availability, improves pore structure, and increases aeration and water permeability. Notably, both microbial fertilizer and green manure applications also demonstrated significant effects in reducing soil pH and electrical conductivity (EC).

Both types of organic fertilizers lowered orchard soil pH (**Table 2**). This phenomenon is closely associated with the acid-base neutralization effects triggered by the microorganisms in microbial fertilizers and the secretion of high concentrations of oxalic and citric acids from green manure roots (Liu et al., 2021; Saeed et al., 2021). This observation aligns with findings by Li et al. (2024b) in their study on different fertilizer management practices in a green manure-maize rotation system (Li et al., 2024). The CM1 and DK1 treatments exhibited the greatest reduction in soil pH, likely attributable to the expanded root distribution and enhanced acidification zone associated with their lower green manure planting densities. Meanwhile, soil EC was also significantly reduced following the application of microbial fertilizer and green manure treatments (**Table 2**). This reduction is primarily attributed to functional microorganisms in microbial fertilizers accelerating the biotransformation of salt ions, reducing NO3^-^ accumulation through pathways like dissimilatory nitrate reduction. Additionally, the organic matter released during green manure decomposition can fix and chelate salt ions in the soil (Zhou et al., 2023; Zhang et al., 2024), thereby decreasing free salt concentrations. Low-density sweet clover proved most effective in lowering soil EC, while both microbial fertilizer and green manure applications led to a significant increase in soil organic matter (SOM) content. (**Table 2**). The core mechanism involves green manure incorporation providing soil microorganisms with abundant, readily available carbon sources, greatly stimulating the microbe-mediated humification process of organic matter (Hu et al., 2023).

Both microbial fertilizers and green manures enhanced the content of soil available nutrients (**Table 2**). Microorganisms in microbial fertilizers and nitrogen-containing precursor substances secreted by green manure roots can stimulate microbial mineralization processes, thereby increasing soil alkali-hydrolyzable nitrogen (AN) content (Pu et al., 2022; Wei et al., 2025). Available phosphorus (AP) showed a significant increase across all treatments. This improvement can be attributed to organic colloids present in microbial fertilizer secretions, which bind with cations such as iron, aluminum, and calcium in the soil, thereby reducing the phosphate ion adsorption capacity of soil colloids. The increase in soil AP is also directly related to the higher acidic phosphatase activity in green manure roots. This enzyme catalyzes the hydrolysis of organic phosphorus, increasing available phosphorus supply (Hummel et al., 2021). Regarding available potassium (AK), all treatments showed significant increases compared to CK. This stems from organic acids produced by microbial fertilizers lowering the microenvironmental pH and disrupting the crystal structure of potassium-containing minerals in the soil, thereby enhancing available potassium content. Green manure facilitates the biological cycling and mobilization of potassium (He et al., 2020). These changes in available nutrients are consistent with the nutrient activation theory proposed for green manures by Amede et al. (2021) (Amede et al., 2021). Low-density sweet clover and oil sunflower showed particularly significant improvements in available nutrients. In summary, planting low-density sweet clover effectively improves the soil physicochemical properties in Korla fragrant pear orchards.

### 4.2 Mineral elements in Korla fragrant pears

Different organic fertilizers exerted varied regulatory effects on the mineral element content of Korla Fragrant Pears (**Figure 1**). Potassium (K) dynamics showed that compared to CK, the JF treatment reduced fruit K content by 2.7%, while the CM1 and DK1 treatments significantly increased it by 12.6% and 16.8%, respectively. This trend exhibited a positive correlation with changes in soil available potassium, indicating a preferential allocation of minerals from green manure to the fruit via xylem transport. Regarding phosphorus (P), however, the JF treatment significantly decreased its content, whereas the DK1 treatment led to a substantial increase (**Figure 1A**). The lack of significant difference in other treatments may be related to the adsorption and fixation characteristics of soil available phosphorus (Shen et al., 2011), highlighting the specificity of P availability influenced by green manure type.

At the trace element level (**Figure 1B**), the CM1 treatment increased manganese (Mn) content. This was attributed to oxalic acid produced during green manure decomposition forming mobile complexes with Mn (Chen et al., 2013). The increase in iron (Fe) content involved green manure residues providing carbon sources for iron-reducing bacteria, promoting the reduction of Fe_2_O_3_ to bioavailable Fe²^+^ (Lyu et al., 2025). By contrast, copper (Cu) levels exhibited minimal variation across the treatments, possibly because acidification promoted the formation of Cu-organic complexes, which in turn constrained its bioavailability (Hu et al., 2024; Li et al., 2024). All treatments significantly reduced zinc (Zn) content (by 34.5-61.9%), with the JF treatment exhibiting the strongest inhibition. Two main factors contribute to this, competitive uptake of Zn by green manure crop roots and fixation through humic acid chelation (Klinkert and Comans, 2020). Grüter et al. (2017) found that long-term combined application of farmyard manure and green manure effectively increased soil zinc concentration. While the application of green manure enhanced wheat yield, the zinc concentration in the grains was not notably improved (Grüter et al., 2017). This suggests that applying green manure alone does not necessarily increase zinc content in all crops; the specific effect depends on the crop species and fertilization practices.

### 4.3 Secondary metabolites

Fruit flavor is closely associated with monosaccharide content. Monosaccharides such as glucose and fructose are primary contributors to the sweetness of fruits, and their levels directly influence the perceived freshness and sweetness of the fruit (Nookaraju et al., 2010). Both microbial fertilizer and green manure applications increased the monosaccharide content in Korla Fragrant Pears, with microbial fertilizer demonstrating a more pronounced effect on fruit monosaccharide accumulation. This enhancement can be attributed to photosynthetic bacteria present in microbial fertilizers, which form symbiotic associations with plant roots. By generating reducing hydrogen and ATP through their own photosynthesis, they indirectly enhance the light reaction efficiency in plant leaves, promoting carbon dioxide fixation and carbohydrate synthesis (van der Heijden et al., 2016). The effect of green manure is closely linked to the restructuring of root-nutrient interaction networks. Green manure treatment increases root biomass and expands the active root absorption zone (Wang et al., 2023), significantly improving nitrogen uptake efficiency and water use efficiency, thereby providing sufficient carbon and nitrogen substrates for sugar metabolism (Wei et al., 2024). Green manure treatment significantly enhances the net photosynthetic rate (Pn) by improving soil aggregate structure, nutrient availability, root development, and chlorophyll synthesis, thereby strengthening the coupling between photosynthesis and sugar transport (Wang et al., 2024). Zhang et al.(2024b) found in studies on tomato fruit nutritional quality that the efficiency of photosynthate transport to the fruit via the apoplastic pathway is enhanced (Zhang et al., 2024) driving up the expression of plasma membrane sucrose transporters (SUT1) (Leister, 2023) ultimately promoting monosaccharide accumulation in sink organs.

Free amino acids, as crucial nitrogen metabolites in plants, play key roles in physiological processes including protein synthesis, regulation of secondary metabolism, and stress response (Galili et al., 2016). Their content directly determines fruit flavor, taste, and nutritional value (Silva et al., 2004). Regarding soil nitrogen bioavailability, enzymes secreted by microorganisms in microbial fertilizers accelerate the decomposition of organic nitrogen, converting it to ammonium or nitrate nitrogen, while promoting nitrogen absorption and transport in the pear roots (Sieradzki et al., 2023). Among green manure treatments, low-density sweet clover and high-density oil sunflower significantly enhanced free amino acid content. The incorporated green manure residues, with their suitable carbon-to-nitrogen (C/N) ratio, activate microbial activity, accelerating the mineralization of organic nitrogen into nitrate and ammonium nitrogen (Mooshammer et al., 2014). Duan et al. (2024) long-term field trial demonstrated that intercropping green manure in tea plantations increased soil ammonium nitrogen, nitrate nitrogen, and available nitrogen by 25.04%, 77.84%, and 48.90%, respectively (Duan et al., 2024). Inorganic nitrogen absorbed through green manure roots is converted to amino acid precursors via the glutamine synthetase (GS) pathway (Xing et al., 2023). Glucose in Korla Fragrant Pears serves as the carbon skeleton for amino acid synthesis. Through the Embden-Meyerhof-Parnas (EMP) pathway and the tricarboxylic acid (TCA) cycle, it generates intermediates such as α-ketoglutarate and oxaloacetate (Guo et al., 2023). These intermediates are direct precursors for synthesizing amino acids like glutamate and aspartate (Liu et al., 2020), further driving the synthesis and accumulation of free amino acids.

Fruit fatty acids act as precursors for aroma compounds and play a critical role in pear flavor formation (Song and Bangerth, 2003). Although esters and aldehydes constitute the primary contributors to pear flavor, fatty acids play a pivotal role in shaping its complexity and uniqueness by modulating the oxidative modification pathways of volatile compounds (Maire et al., 2013). This study found that different microbial fertilizer and green manure treatments significantly modulated the fatty acid composition in Korla Fragrant Pear fruit. Green manure, particularly low-density sweet clover and high-density oil sunflower, significantly enhanced fruit fatty acids. This effect correlates with the capacity of photosynthetic bacteria present in microbial fertilizers to enhance leaf chlorophyll content and photosynthetic enzyme activity in pear trees. This enhancement improves carbon fixation efficiency, thereby generating increased levels of photosynthetic assimilates, such as glucose. Glucose is converted via glycolysis to pyruvate, which is then transformed into acetyl-CoA, the fundamental precursor for fatty acid synthesis (Shi and Tu, 2015). Sieradzki et al. (2023) found in studies on chickpea growth and yield under drought that applying microbial fertilizer containing plant growth-promoting rhizobacteria, including photosynthetic bacteria, significantly increased leaf chlorophyll content (Sieradzki et al., 2023). Green manure input is associated with the upregulation of fatty acid desaturase FAD2/3 activity (Jin et al., 2024). From a metabolic source perspective, plant fatty acid synthesis predominantly depends on the acetyl-CoA carboxylase (ACCase) system localized within chloroplasts and plastids (Zhou et al., 2024). Furthermore, green manure cultivation significantly increases photosynthetic carbon assimilation efficiency in pear tree leaves by enhancing soil nutrient availability and soil β-glucosidase activity (Khan et al., 2025). The increase in photosynthates not only provides ample acetyl-CoA substrate for fatty acid synthesis (van Rossum et al., 2016) but also upregulates the expression of the BnFAX6 gene via sugar signaling pathways, thereby promoting lipid synthesis (Huang et al., 2021).

### 4.4 Volatile organic compounds

Zinc (Zn) deficiency disrupts volatile organic compound (VOC) synthesis through a dual mechanism. Firstly, as an essential cofactor for numerous enzymes such as alcohol dehydrogenase (ADH, EC 1.1.1.1), Zn deficiency likely directly reduces enzymatic activity (Dalziel, 1963). Compared to the CK treatment, all organic fertilizer treatments (JF, CM1, CM2, DK1, DK2) resulted in a significant reduction in total volatile content in Korla Fragrant Pears, with aldehydes decreasing by 1.40-60.08%. Reduced Zn content in pears diminished ADH activity, thereby impairing the oxidation of alcohols to aldehydes. Consequently, the conversion of 1-hexanol to hexanal was inefficient, leading to significant reductions (ranging from 2.45% to 61.46%) in aldehydes such as hexanal and nonanal. ADH is intrinsically reversible, catalyzing both the oxidation of alcohols to aldehydes (forward reaction) and the reduction of aldehydes to alcohols (reverse reaction) (Ratautas et al., 2017). When Zn²^+^ deficiency lowers ADH activity, the reverse reaction efficiency decreases more significantly, hindering the conversion of aldehydes to alcohols and resulting in a 14.80-77.32% decrease in alcohol content.

Secondly, Zn is an integral component of the active site of copper-zinc superoxide dismutase (Cu/Zn SOD, EC 1.15.1.1) (Wada et al., 2013). Its deficiency reduces Cu/Zn SOD activity, leading to reactive oxygen species (ROS) accumulation (Tian et al., 2021). Additionally, arginine (Arg) deficiency (measured via HPLC-UV; content reduced by 14.29–28.57%) leads to ROS accumulation. This occurs because Arg is converted by arginine decarboxylase (ADC) into putrescine and subsequently polyamines, which function as ROS scavengers. Consequently, ROS accumulation inhibits lipoxygenase (LOX)-catalyzed oxidation of linoleic acid, thereby reducing the production of aldehydic flavor compounds such as decanal (Pasquariello et al., 2015). On the other hand, ROS may accelerate the non-enzymatic peroxidation of residual aldehydes (e.g., decanal) to carboxylic acids (e.g., decanoic acid), exacerbating flavor loss. Studies in apricots have confirmed that increased LOX activity promotes the accumulation of C6/C9 aldehydes, enhancing “green aroma” (Sun et al., 2024). Conversely, attenuated LOX activity weakens the linoleic/linolenic acid oxidation pathway, reducing the generation of green leaf volatiles like 2-hexenal (decrease of 7.03-55.59%) (Chen et al., 2020), and causing the accumulation of linoleic and linolenic acids (increases of 86.5% and 162.5%).

When Zn is deficient in the fruit, mitochondrial membrane disorganization leads to reduced electron transport chain (ETC) efficiency and increased electron leakage. This results in massive accumulation of ROS such as superoxide anion (O_2_^-^) and hydrogen peroxide (H_2_O_2_), triggering oxidative stress (Gupta et al., 2024). Elevated ROS levels directly target key functional domains of the pyruvate dehydrogenase (PDH) complex, with the lipoic acid cofactor being a primary site of oxidative attack. This results in the oxidative inactivation of lipoic acid (Lee and Kim, 2024) and impairing PDH’s ability to catalyze the conversion of pyruvate to acetyl-CoA (Lei et al., 2024). Acetyl-CoA is an essential precursor for ester synthesis (Shi et al., 2021). Its deficiency directly limits the biosynthesis of esters (e.g., butyl acetate, isoamyl levulinate), leading to a 14.31-59.47% decrease in total ester content.

Pearson correlation analysis revealed an inverse relationship to the promoting effect of Zn on VOCs. Manganese (Mn) and iron (Fe) elements showed significant negative correlations with most volatile compounds (r = -0.34 to -0.98). Mn excess competitively inhibits ZIP family transporters, reducing Zn bioavailability (Shanmugam et al., 2011). Under Fe²^+^ overload and H_2_O_2_ accumulation, excessive ·OH radicals are generated through the Fenton reaction within fruit tissues, inducing oxidative damage to aroma precursors (Yin et al., 2024). The interaction of these two elements may also interfere with the jasmonic acid (JA) signaling pathway, subsequently downregulating the expression of LOX and alcohol acyltransferase (AAT) genes.

The significant reduction in fucose content in Korla Fragrant Pears amplified the negative effects of Zn deficiency. As a core component of pectic polysaccharide rhamnogalacturonan II (RG-II) (Waszczak et al., 2024), a concurrent 20% decrease in tyrosine (Tyr) levels interferes with phenylalanine hydroxylase activity, indirectly affecting the generation of lignin precursors like coumaric acid (Zhou et al., 2021). Deficiencies in fucose and tyrosine (Tyr) compromise cell wall integrity, resulting in widened escape channels for volatile compounds. This structural alteration increases the susceptibility of aldehydes and esters to both volatilization and enzymatic degradation by oxidative enzymes (Muller et al., 2009). Together, these factors constitute a cascade regulatory network described as “Zn deficiency - metabolic imbalance - oxidative stress - weakened defense”.

In conclusion, the decline in soil Zn availability induced by microbial fertilizer application and green manure cultivation is a key limiting factor responsible for the reduced synthesis of characteristic VOCs (particularly aldehydes, alcohols, and esters) in Korla Fragrant Pears. The underlying mechanism likely involves the inhibition of key enzyme activities such as ADH and SOD, consequently affecting metabolic pathways including fatty acid oxidation, alcohol-aldehyde conversion, antioxidant balance, and acetyl-CoA supply.

## 5 Conclusion

Organic fertilizers synergistically improve the core nutritional parameters of Korla Fragrant Pears by optimizing the soil microenvironment and regulating metabolic networks. Specifically, the application of microbial fertilizers combined with green manure cultivation significantly lowered orchard soil pH, increased soil organic matter content, and enhanced soil electrical conductivity, collectively reshaping soil physicochemical properties. These practices also significantly promoted the accumulation of monosaccharides, fatty acids, and free amino acids in the fruit. Microbial fertilizer application was particularly effective in enhancing fruit monosaccharide content, while low-density sweet clover (CM1) and high-density oil sunflower (DK2) treatments were most effective in increasing fruit fatty acid and free amino acid content. However, green manure cultivation induced a decrease in soil zinc (Zn) availability. This initiated a cascade effect that suppressed key flavor-related metabolic pathways, markedly hindering the biosynthesis of volatile alcohols, aldehydes, and esters. Consequently, the overall concentration of volatile organic compounds (VOCs) declined, ultimately attenuating the fruit’s signature aromatic profile. In summary, to fundamentally enhance the core nutritional quality of Korla Fragrant Pears and improve soil fertility, the optimized sweet clover (CM1) green manure strategy is recommended. Nevertheless, to balance the improvement in basic nutritional quality with the maintenance of characteristic flavor (compromised due to inhibited VOC synthesis), it is imperative to concurrently strengthen Zn nutrition management when implementing green manure cultivation. This integrated approach is essential for effectively promoting the sustainable development of the Korla fragrant pear industry.

## Data Availability

The raw data supporting the conclusions of this article will be made available by the authors, without undue reservation.
